# What Clinical Pharmacology Means To Us

**DOI:** 10.4103/0973-1229.27614

**Published:** 2006

**Authors:** S Malhotra, N Shafiq

**Affiliations:** Department of Pharmacology, Postgraduate Institute of Medical Education and Research, Chandigarh, India

**Keywords:** *Clinical Pharmacology*, *Me-Too Drugs*, *Negative Studies*, *Informed Consent*, *Research and Future of the Branch*, *Ethics and Pharma Relation*

## Abstract

Clinical Pharmacology is a specialty with many attributes and our association with the subject has allowed us to acquire, apply and disseminate myriad aspects of research and practice. Though clinical pharmacologists are conspicuous by virtue of their small number, recent years have shown a growing need for the course. In the review below we navigate through several aspects of the subject as we encountered them from time to time. From critical appraisal of literature, to application of knowledge of drugs, to clinical practice; moving on to clinical and basic research, to drug development process, to policy making - these are but a few of the many fields which constitute the scope of clinical pharmacology. The importance of the subject lies in allowing a trainee to develop a broad overview of the entire process, from drug generation to drug distribution to drug utilization, a process meant for the greater common goal of better health for all. We foresee a bright future for the subject though with a slight skepticism thrown in. In the present article, we make use of personal experiences and reference from literature to help you get a broad view of what clinical pharmacology means to us.

## Introduction

### My Name Is Clinical Pharmacology

Though it is not customary for authors of invited write-ups to introduce themselves, we would like to digress slightly from the norm. We make use of our professional degree for describing ourselves, and hence Clinical Pharmacologists are what we are. In case some of the readers are bemused by this description, they need not feel guilty of ignorance, for we indeed belong to an endangered species. This description is originally not ours and has been borrowed (Vestal, 1998). Unlike our other super speciality brethren such as cardiologists, gastroenterologists, nephrologists etc who are virtually omnipresent, we are conspicuous by our near absence. While at the international level this means concerns regarding lesser residents opting for clinical pharmacology (Benowitz, 1997), at the national level the concern is regarding aborted attempts at opening clinical pharmacology centers.

Having collectively spent a very enriching period of more than a decade in this field, we have moved from being trainees to teachers of Clinical Pharmacology. However, we were learners then and are learners still. Every now and then some new concept emerges. And in the process of grasping it, teaches us repeatedly that ‘becoming veterans from amateurs’ is a Herculean task. All the same, it is heartening to know that the super speciality is beginning to find its place in such gatherings. We certainly believe in the chaos theory and the butterfly effect propounded by Edward Lorenz. Using Ian Stewart’s words (Stewart, 1989):

The flapping of a single butterfly’s wing today produces a tiny change in the state of the atmosphere. Over a period of time, what the atmosphere actually does diverges from what it would have done. So, in a month’s time, a tornado that would have devastated the Indonesian coast doesn’t happen. Or maybe one that wasn’t going to happen, does.

We were fortunate to have found an academic accommodation in one of the institutes where D.M. in Clinical Pharmacology course was the first of its kind in India. More importantly, it did not demonstrate a considerable lag from the time Clinical Pharmacology got its official birth date from WHO in 1970 *(World Health Organ Tech Rep Ser.*, 1970). The doyens of the subject, Prof. Ranjit Roy Chaudhury, Prof. P.L. Sharma, Prof. V.S. Mathur, and several others, actually deserve a lot more than mere mention for their foresight, vision and dedication, and for the fact that they continue to guide us till date.

A very commonly asked question by our peers on learning that we were clinical pharmacologists used to be, ‘Did you take up this subject out of choice?’ The question is a good reflection on the choice of clinical pharmacology as a speciality. Most of the people who opt for the branch have majored in pharmacology, which, being a para-medical branch, was not a coveted subject for those making it through the postgraduate entrance exams. There has been a very interesting trend of late, though. Students who score well in the exams and are among the top rankers are beginning to opt for the subject. Till a few years back we thought it was a chance happening. However, the trend has continued to stay and we believe it is *clinical* pharmacology that is making pharmacology more attractive. Two important reasons for this change are lucrative and challenging job prospects in the pharmaceutical industry (though personally this is not our favoured reason), and increasing awareness of the diverse scope of this subject.

Besides being our bread and butter, the subject has meant a great deal to us. We like to see it best as the bridge between basic science and clinical science (cynics tell us we are neither pharmacologists nor clinicians!): a path from bench to bedside. This bridge allows application of the available knowledge in patient care and policy making, and it also helps in generation of knowledge mainly for these two purposes.

Now this quick summary of what it means to us warrants some elaboration. We will do that by taking you through some selective examples that have gone on to become our experience in clinical pharmacology

## Clinical Pharmacology A Path From Print To Bedside

As part of training during our posting in the Department of Internal Medicine, we were asked to review the prescription of patients and comment.

**Case 1:** We had a case where the patient was referred to the emergency department for haemorrhagic stroke. The medicine resident presented findings and we were asked to comment on the possibilities. Our tuning with clinical pharmacology compelled us to take the medication history. The patient had been thrombolysed with streptokinase for myocardial infarction, which had preceded the cerebrovascular accident. Having undertaken a cursory causality assessment for the adverse drug event we categorized it as ‘probable’. We were immediately asked to comment whether the option of thrombolysing the particular patient was appropriate. This meant that we check for all the contraindications for using a thrombolytic agent, which is what we did, and ruled out the possibility of an irrational use of the agent. This was not the end of the story as, following our answer, we were asked:

If seeing this patient, another well informed attendant of a patient of myocardial infarction asks, ‘Is it really necessary to thrombolyse his relative?’ what would our answer be?” In other words, “What is better- to let him have the pain or to lead him to a near paralytic state?

Inadvertently we had treaded into the territory of evidence-based medicine when we formulated our answer and told the patient’s relative:

Streptokinase reduces the risk of mortality to 6.3% as against previous 13% (The GUSTO Investigators, 1980). For each hour earlier that a patient was treated, there was decrease in absolute mortality by 1% that translated into an additional 10 lives saved per 1000 patients treated (Michel and Weinfield, 2000). There are some concerns regarding haemorrhagic stroke. These are minimal. The risk of intracerebral haemorrhage is approximately 0.3% (Michel and Weinfield, 2000). Weighing the risk and benefit we advocate the thrombolysis of your patient. Moreso since you have reached the patient to the emergency within three hours, the expectation of benefit is maximum.

We realized later, at the end of the round, that clinical pharmacology was beginning to find its entry into our veins.

## ‘Me Too’ Drugs

There are several other aspects while writing a prescription that one is impelled to consider. Important among them being a burgeoning of ‘me too’ drugs which offer no clinically relevant advantage over the progenitor drug. The pharmaceutical companies are able to bring and later push them into the market reaping colossal benefits. An example is the case of Astra Zeneca’s Nexium, which was brought into the market just as Prilosec, another Astra Zeneca product, was going off patent. The drug hardly offers any clinical advantage over Prilosec or, for that matter, any generic version of Omeprazole.

It is often argued that ‘me-too’ drugs offer an opportunity for reduction of patented drugs by bringing down the price. This may be an exception rather than a rule as it has been noted that the ‘me-too’ versions are introduced, if not at a higher, at an equal price.

What does one do, now that ‘me-toos’ have become a non-ignorable reality? It is a prudent exercise for a person to devise a personal formulary based on availability, cost, ease of administration, side-effect profile, drug interactions, consideration for route of elimination and choose the most suitable option for any given condition. Of course, the formulary should offer enough flexibility to suit certain special situations. Training in clinical pharmacology is indeed a great facilitator in converting such ideas into routine practice.

## Clinical Pharmacology: From Bench To New Drug Use

The second episode that we would like to tell you about helped our foray into yet another territory of clinical pharmacology.

**Case 2:** In one of our informal discussions on pleiotropic effect of some drugs, we chanced upon an article, which related the antiproliferative and anti-inflammatory action of troglitazone, an anti-diabetic drug in an experimental model of psoriasis (Ellis, Varani, Fisher, Zeigler, Pershadsingh, Benson, Chi and Kurtz, 2000). Troglitazone had by then been withdrawn but another agent from the same class, pioglitazone, demonstrated similar properties. We were immediately motivated to initiate a pilot study of pioglitazone in patients with plaque psoriasis, a condition characterized by derangements in dermal proliferation and inflammatory processes. We wrote down the protocol and finalized it in consort with the dermatologist. A brief tryst in a pharmaceutical company allowed us to manage the drug and matching placebo. We drafted the CRF, generated a randomization list, developed Standard Operative Procedures for various things and conducted the study in accordance with the principles of GCP, and with funds just enough for the stationery. The study showed a moderate improvement in Psoriasis Activity and Severity Index (PASI) at 10 weeks, which was the primary outcome (Shafiq, Malhotra, Pandhi, Gupta, Kumar and Sandhu, 2005a).

Being impoverished on resources we could not hire a biostatistician for the analyses. So we carried out the statistical analyses ourselves. The findings of the study warranted some additional investigations. We have presently undertaken a study to evaluate the efficacy and safety of combining acitretin with pioglitazone.

So we were beginning to grow with the subject itself, exploring its various domains.

## Clinical Pharmacology: Sifting The Real From Projected Truth

The very same discussion on pleiotropic effects of drugs motivated us to explore the available literature on this issue related to statins.

**Case 3:** The database was really vast and we realized statins were implicated in almost everything. They seemed to have potential, or almost proven, role in conditions ranging from sickle cell anemia to rheumatoid arthritis to Alzheimer’s disease. When some of our better-informed colleagues had started to believe that mixing statins in the drinking water supply might well be justified, we thought of taking a closer look. What lay in store for us was not a pleasant surprise but exaggeration of facts, distortion of reality and overgeneralization of study findings. For instance, statins were investigated as potential beneficial agents for multiple sclerosis, on account of their anti-inflammatory properties. The title of the study ran as follows, ‘Oral simvastatin treatment in relapsing-remitting multiple sclerosis’ (Vollmer, Key, Durkaisiki, Tyor, Corboy, Markovic-Plese, Preiningerova, Rizzo and Singh, 2004). The investigators evaluated beneficial effect of 80 mg of oral simvastatin for 6 months on gadolinium enhancing lesions. The study was an open label study in which the posttreatment lesions were compared with baseline lesions and simvastatin treatment was shown to have beneficial effect. The results of the study need to be interpreted with caution as there existed a definite possibility that reduction in the disease severity as measured by MRI may very well have been due to regression to the mean. Moreover, the patient selection on the basis of gadolinium enhancing lesion may have led to selection of patients with active disease. These patients were likely to have shown a spontaneous reduction in the disease severity anyhow. Steroid use and unblended assessment of the MRIs were additional factors that could have influenced the study results.

Similar close look at several other proposed indications of statins showed that the evidence was a rather weak one. Even for the conditions for which randomized controlled trial results were available, the beneficial effect had been shown either on a surrogate marker or, in several cases, had not taken care of the several biases in drawing conclusions. We were compelled to wonder, ‘Statinth wonder of the world: a panacea for all or a bubble about to burst.” (Shafiq, Malhotra, Pandhi and Grover, 2005 b). The review after being rejected by a few journals found a place in the *Journal of Negative Results in Biomedicine* (*www.jnrbm.com/content/4/1/3*).

We understand that now more and more people are realizing the importance of publishing so called ‘negative’ studies which should more appropriately be studies showing a lack of statistically significant beneficial effect (Malhotra, Shafiq and Pandhi, 2004a). There are journals, for example, *Journal of Negative Results in Biomedicine (www.jnrbm.com/home/)* that are devoted exclusively to such studies. What was happening earlier was that only studies showing a breakthrough, or which showed p< 0.05 for one or more of the study outcomes, found their way into medical journals. As a result many scientists, who were perhaps guided by factors other than true scientific pursuit, were tempted to forge results and receive not only accolades from their peers but also flaunt better citations. Then again, at the time of doing meta-analysis, ‘negative’ studies were conspicuous by their absence, and for many years the disturbing statement, ‘Publication bias cannot be ruled out,’ remained.

There have been several instances when investigators have been discouraged (and even sued) by sponsors for publishing results that may not have been fruitful for a company. The role a sponsoring pharmaceutical company may play in moulding the direction of a research work cannot be ignored. In one study (Stelfox, Chua, O’Rourke and Detsky, 1998), the authors scrutinized the conflicts of interest issues associated with publications of calcium channel blockers. The authors identified 70 studies and classified them as critical (23), supportive (30) or neutral (17%). They then queried the authors for financial relationships with manufactures and found that 96% of those who supported the study had financial interests with manufactures of calcium channel blockers. From among those who were either critical or neutral, 37% and 60% respectively had such financial interests. This may just be a case in point.

## Clinical Pharmacology:A Conscientious Viewpoint

Perhaps no other speciality is as intimately associated with the pharmaceutical industry and drug regulatory authorities as is clinical pharmacology. Not only has the branch and its older sibling, ‘Pharmacology,’ been the main source of recruitment of personnel for the pharmaceutical industry, those staying on become the industry’s greatest critics as also collaborators.

A good grounding in clinical trial methodologies, critical evaluation of literature, concepts of patents and generics, statistics, pharmacovigilance allow many clinical pharmacologists to read between the lines and correct interpretation of published reports of sponsored studies, of the role of ‘conflicts of interests’ in obtaining drug approval or withdrawing a drug, thus making them the pharma industries’ biggest critics. The same repository of knowledge also allows them to become a part of the industries’ endeavour for new drug development by carrying out and interpreting their experimental research, their toxicological studies, and conducting clinical trials either as employees of the companies or as members of academic institutes.

We have long been critical of the ways pharmaceutical companies promote their products. Some Greco-Latin versions of these irrational promotional principles have been pointed out *(see www.nofreelunch.org/ slide.htm): ‘Argumentum ad Verecundiam,’ ‘Argumentum ad Populum,’ ‘Non Sequitar,’ ‘Argumentum ad Misericordiam,’* translated into English ‘appeal to authority,’ ‘the bandwagon effect,’ ‘the red herring,’ and ‘appeal to pity’, respectively (The Physician-Pharmaceutical Industry Relationship, 2006).

The point, which was largely ignored, was the fact that this is possible only because the clinician writing the product is up for sale. It is just a matter of bargaining. One of our surveys regarding the perception of pharmaceutical company representatives brought to light some alarming facts (Malhotra, Kondal, Shafiq, Sidhu and Pandhi, 2004 b). It was noted that pharmaceutical company representatives were of the view that they were able to influence more than half of the prescriptions. Gifts, sponsorship of conferences and provision of scientific literature were helpful in getting a desired drug promoted. We thus decided to at least train the clinicians in critical analysis of such promotional literature at various low profile workshops.

Drug regulators have several important issues to handle. In developed nations, approval and disapproval of innovator molecules is the major issue with regulators. In developing countries, other involved issues are - procurement and distribution of medicines, import of drugs, formulation of guidelines for conducting clinical trials, dealing with the problem of the dearth of ethics committees in a large number of health centers, and a relative lack of awareness of GCP principles. Regulatory authorities are often at the receiving end for being under the influence of the pharmaceutical industry, and many times rightly so.

A glaring example of the same brought to light by action taken by the Arthritis Advisory Committee and the Drug Safety and Risk Management Advisory Committee meeting held in February 2005, deserves special mention here. Safety of COX-2 inhibitors was the topic of discussion. It was disclosed after the meeting that 10 of the 32 voting panel members had financial associations, in some form or another, with the manufacturers of the COX-2 inhibitors. Of the 30 votes cast by these 10 members on whether rofecoxib, celecoxib, and valdecoxib should continue to be marketed, 28 favoured marketing of the drugs. Of the 66 votes of the other 22 members, only 37 favoured their marketing. If the 10 panel members with the financial associations had not participated, the committee would have voted 12 to 8 that valdecoxib should be withdrawn and 14 to 8 that rofecoxib should not return to the market. However, with these voters being there larger than life, the tallies were 17 to 13 for keeping valdecoxib on the market and 17 to 15 for the return of rofecoxib (Harris and Berenson, 2005; Center for Science, 2006). Such was the impact of the ‘Godfather’ that Dr. Wood of Vanderbilt University, who had chaired the joint meeting, commented:

Of all the FDA advisory committee meetings I have attended, there has never been more money on the table. Some potential panel members had already been excluded because of conflicts. The people who were chosen had disclosed their financial interests to the FDA, although it played out as though they had something to hide (Steinbrook, 2005).

Heavy conflicts of interests of members in the drug approval reviewing committee are a major cause for concern. Though the Declaration of Helsinki *(http://www.wma.net/e/policy/b3.htm)* outlined ethical principles for the conduct of research in humans several decades ago, clinical trials defying the ethical principles continue to occur in modern times.

Two very contemporary examples, which are perhaps more blatant in flouting ethical norms than many others, are given here:

1. A study conducted by a pharmaceutical company in an African country for perinatal transmission of HIV deprived the control group of an established treatment of proven efficacy. The control group was given placebo on the pretext that ‘no treatment’ for preventing perinatal transmission was the norm in the native population (Lurie and Wolfe, 1997).
2. The other example hails from one of the more affluent and aware zones of the world. A study to elucidate the natural history of cervical cancer was conducted in Australia. Here again the women were deprived of the benefit of regular screening (which has already shown to have a definite role in detecting early stages of cancer) in order to update the existing knowledge on cervical cancer (Coney and Bunkley, 1987).

Despite such examples continuing to crop up from time to time, it is heartening to note that, in general, awareness regarding ethics in medical research is ever increasing. Developing countries are also taking initiatives to propagate the concept of ethics by organizing workshops, seminars, helping institutes set up ethics committee and issuing their own guidelines. Guidelines for ethical conduct of human research, issued by Indian Council of Medical Research are oft quoted in this regard *(www.icmr.nic.in/ethical)*. The recent Academia-Industry Symposium by the *Mens Sana Monographs* is a welcome step in the direction of creating a healthier climate for interaction between the medical profession and the pharmaceutical industry (Singh and Singh, 2005a; 2005b; 2005-2006. See also *http:// mensanamonographs.tripod.com/id87.html* for details*.)*

Rightly, clinical pharmacology training here and abroad lays a great deal of emphasis on the ethics of conducting research in humans. For the clinical pharmacologists involved in bench side research, training in ethical principles of conducting research in animals is of as much importance as conduct of the research itself.

## Informed Consent

In developing nations, the importance of conducting clinical trials is being increasingly recognized. There is more than one issue which needs attention in this regard. While conducting these studies we realized obtaining informed consent was a rather difficult job. The main reason was that participants held two kinds of extreme viewpoints about clinical research: one, that they would become guinea pigs; or, second, that of leaving every decision to the investigating physician (expressed in the local dialect as, *‘Jo tussi theek samjho’,* translated roughly as, ‘Whatever you decide is fine with us.’).

It was interesting to note that till very recently, even in tertiary care centers conducting multi-centric trials, the importance of obtaining truly informed consent in the vernacular was nothing more than an interesting topic for discussion. Fortunately, though, the trend is changing. Having realized the importance of the same, we started an exercise in designing a template consent form (Shafiq, Sidhu, Pandhi and Malhotra, 2005 c). This template is based on research in which we have tried to assess the comprehension of research subjects after giving them a dummy consent form. Interestingly, despite explaining, both in writing and orally, terms like ‘placebo’, ‘randomization’ and ‘blinding’ were not largely understood even by the educated. After realizing this, we have started an exercise in which we allow the patient to ask questions on whatever they have not understood, and continue to explain it to them till it is clear. In one of the meetings of investigators for an ongoing trial, our colleagues in other institutes were happy to know that we had maintained a record of patients refusing consent, with reasons for the same. As an experimental exercise, we make a note of the time the consent process lasted and the queries that were raised.

There were several other important misconceptions prevailing both in the patients as also the clinicians who participate as investigators in a clinical trial. The need for educating people regarding this was imminent. We have made an attempt in this direction by penning down differing aspects of clinical research which may be of concern to a clinical research participant in the form of a book written in a simple language (Malhotra, Shafiq and Pandhi, 2006). We are yet to see how effective our initial attempt will be in the years to come.

## Clinical Pharmacology:From Ignorance To Knowledge

Another episode we would like to recount here is about low molecular weight heparins.

**Case 3:** We had long been hearing about the superior efficacy of the low molecular weight heparin, enoxaparin, over its other counterparts -nadroparin and dalteparin. In our earlier investigation we had seen that enoxaparin, a low molecular weight heparin (LMWH) showed a benefit over conventional heparin therapy on the composite endpoint of death, recurrent angina and myocardial infarction (Malhotra, Bhargava, Grover, Pandhi and Sharma, 2001). It was now time to investigate the best choice among the three available to us in the Indian market for treating unstable angina.

We thus concluded there was an urgent need for a head to head comparison among the three most commonly used low molecular weight heparins in patients with unstable angina. We decided to compare the efficacy, safety and cost-effectiveness of enoxaparin, nadroparin and dalteparin in patients with unstable angina. As a sub study, we decided to investigate the effect of these LMWHs on Plasminogen Activator Inhibitor-1. Our results led to the following conclusions - the three LMWHs were similar as regards efficacy and safety. The cost minimization analysis showed nadroparin to be superior to dalteparin and enoxaparin. We thus concluded that cost of the LMWH should be the determining factor for making a choice of the LMWH (Unpublished data).

The perspective of the pharmacoeconomic study was the patient. Unlike in the West, patients here in India bear majority of the treatment cost. Barring few exceptions, they are largely uninsured. Obtaining an indigenous cost effectiveness data is, therefore, the need of the hour. This then becomes the basis for further prescriptions being written by practicing physicians.

## Clinical Pharmacology: Making Its Heartbeats Felt

By this time the busy clinicians have started understanding the different approach of a clinical pharmacologist in answering several drug and therapeutics related questions. The need for generating local treatment guidelines had long been realized by our fellow clinicians. The first such guideline, which was generated in the institute by collaborative effort of WHO, had largely overlooked clinical pharmacologists (Jindal, Gupta and Aggarwal, 2006). In the generation of their next guideline for the management of community-acquired pneumonia, clinical pharmacologists were invited as a part of the team of experts (Guidelines to be published).

Then, again, comes the question of interpreting clinical guidelines made by ‘experts’ in the more privileged parts of the world till one has one’s own guideline ready. In the present era of evidence-based medicine, a prescription that does not follow the Joint National Commission guidelines, more popularly known as JNC guidelines, for the treatment of hypertension may well be questioned. However, blindly following it would also mean ignoring several important points, such as the rather dogmatic recommendation of diuretics as first line management, introduction of the term ‘prehypertensive’ and the conflict of interests of the people involved in generation of the guidelines (Shafiq, Malhotra and Pandhi, 2003).

Again there was a suggestion sometime back of polypill therapy for the primary and secondary prevention of cardiovascular diseases. The authors suggested combining thiazide diuretic, angiotensin converting enzyme inhibitor, beta-blocker, statin, aspirin and folic acid for this purpose. The suggestion was based on the synergistic treatment effects calculated by multiplying relative risk reductions with each of the individual therapies (Wald and Law, 2003). Though it sounded interesting, if allowed to go unchecked and unquestioned, it may have resulted in the pharmaceutical industry grabbing the seemingly attractive opportunity of pushing such a pill into the market. Fortunately, though, such writeups are now under the scanner, and many a time voices raised are successful in critically evaluating them.

It was pointed out that utility of individual pills for primary prevention could not be generalized (Fahey, 2005). The example of aspirin was cited in this context wherein it was pointed out that low dose aspirin did not provide any benefit in prevention of rate of myocardial infarction in women as was the case in men (Fahey, 2005). It was also argued that treating people with low risk of cardiovascular diseases as was suggested by the authors would lead to medicalization of the population with no promise of sufficient benefits coming through (Mulrow and Kussmaul, 2005).

## Clinical Pharmacology: Research Sans Frontiers

The members of our species have two major options for diversifying after training. They usually make a choice between either joining a pharmaceutical industry or doing academics. These two have often been seen as mutually exclusive, or rivals.

Incidentally, the clinical pharmacology community is rather a small one in India. The small size has made it a closely-knit community, with of course all its internal badgering and dissensions. But it has also helped us evolve reasonably decent academia-industry collaboration rather than an academia-industry rivalry. Many company sponsored trials and bioequivalence studies are outsourced to us with due credit to clinical pharmacology. Other than providing us with much needed financial support, this has provided us hands on experience in learning to undertake such studies. On our part, we have been conducting national workshops in Clinical Pharmacology yearly, and recently have initiated, with industry support, a workshop for training in Good Clinical Practice. We have plans of collaboration with the pharmaceutical industry to generate a core group of clinical researchers who would train other academicians for conducting research. As a routine, we write protocols for investigator-initiated trials and approach concerned industry to sponsor our study in terms of provision of drugs, placebo, or the comparator and other requirements. To finalise such protocols, we have experts sitting from our department, the concerned clinician, and a person from the pharmaceutical industry.

From that time on, a very good rhythm is established as the study moves from initiation to completion to analysis to publication.

Though the pharmaceutical industry is largely maligned for malpractices, we have had instances where we have gone ahead with publishing results that may not have been very welcome to the industry. This study aimed at comparing kinetics of a new iron formulation (iron polymaltose complex) with the older ferrous sulphate formulation. The manufacturers of the new formulation sponsored the study. However, the study results showed a greater bioavailability of ferrous sulphate as compared to the new formulation. The study results were published as such (Malhotra, Garg, Khullar, Malhotra, Kondal, Rana and Sidhu, 2004).

Interestingly, our relationship with the concerned person never got jeopardized: instead what happened was rather to the contrary.

The foundation of a good working relationship between the two has led us into more such joint ventures.

## Clinical Pharmacology: The Help Line

We steer you through another scenario that has been a part of our day to day living.

**Case 4:** A phone call comes to the drug information unit located in our institute. It is a query from a private dermatology clinic. A female patient of the dermatologist had used dextromethorphan, Augmentin and flucanozole within the first five weeks of pregnancy. The dermatologist wanted to know if it was advisable to continue the pregnancy.

The resident on call, though a fresh postgraduate student, was tuned to clinical pharmacology. He reported, ‘High doses of fluconazole in pregnancy cause teratogenicity in animals’. He cited a case report of craniofacial abnormality in the foetus of a mother who had taken 400 mg of fluconazole daily (Pursley *et al*, 1996). It was added, further, that prescription event monitoring found no such increase in teratogenicity but reports of teratogenicity with high dose fluconazole use were available. Cardiac abnormality had been observed in females taking high doses during pregnancy. However, dextromethorphan and Augmentin have not been shown to have any teratogenic effects (Brunton, Lazo and Parker, 2006).

These findings were presented to the dermatologist who in turn conveyed it to the parents. So services provided by a clinical pharmacologist come handy in multiple ways.

## Roadblocks And The Future

Though we see a bright future for the subject, we also foresee a number of roadblocks. For instance, at the moment, there are only a limited number of institutes imparting the degree. More importantly, the number of trained faculty at these institutes is even fewer. Funds for more such centers are slow to come by.

However, with the government and pharmaceutical industry showing renewed interest in the subject, the pattern is likely to change. Judicious use of drugs, development of new formulations, reaching treatment to all concerned is no longer confined to discussion by the intelligentsia, but is being felt as the need of the hour. The ‘10-90’ gap (ten per cent of worldwide expenditure on health research and development is devoted to the problems that primarily affect the poorest 90 per cent of the world’s population) is increasingly being seen as a reason of some serious action. (In this connection, see *http://www.who.int/intellectualpropery/submission/InternationalPolicyNetwork.pdf.)*

The clinical pharmacologist, sitting at the hub of all drug related activities, is the right person to lead the team of individuals getting together to deal with this inequation. While the optimistic picture presented above speaks of events in the next two or three decades at best, a time is bound to come when unmet medical needs will remain countable on fingertips.

What will be the future of clinical pharmacologists in the next half of the century may send shudders down the spine of quite a few. Fortunately, though, the human body, with its ever-unfolding mysteries and aspects, has been able to keep alive the need for research in therapeutics so far. But like other sciences, the focus may shift from research in ischaemic heart diseases to anti-ageing medication, to treatment for male pattern baldness, or one-time injections for rheumatoid arthritis.

On second thoughts, leads are slow to come in certain fields, and clinical pharmacology may guide and accompany humanity towards a better and healthy living, what with quality of life studies already becoming an integral part of research (Shah, Ananth, Sohal *et al*, 2006; Bradshaw, Jamrozik and Gilfillan, 2006).

A few concerns remain, such as how to increase the number of institutes who provide clinical pharmacology training. Most of the ongoing talk is about incorporating clinical pharmacology into the undergraduate curriculum. Indeed, orientation towards some concepts of clinical pharmacology could start at the undergraduate level. For instance, teaching clinical application of pharmacokinetic knowledge would be more appropriate than making it into a boring exercise of rote learning of formulae. Again, it would be more prudent to help students generate their own formularies based on the available drugs for a particular condition. Also, training in searching for evidence for any fact mentioned in their textbooks could be a good exercise. This should pave the way for generating a greater motivation in students to opt for clinical pharmacology later.

The paucity of institutes giving training in clinical pharmacology at the super speciality level is an important issue. Even if more such institutes were opened, absence of good trained faculty would be another roadblock. More important would be retaining the faculty in academic institutes, as the lure of pharmaceutical industry pay packets is a great force to reckon with. Of late, some pharmaceutical companies have started their own courses in clinical pharmacology. However, these courses deal with only some aspects of the subject, such as clinical trials, not clinical pharmacology in totality. The importance of the subject lies in its capacity to integrate translational and clinical research.

Though clinical pharmacologists have been providing good insights into several drug related issues, such as rational use of drugs, essential drugs list, pricing, regulatory issues, the branch *per se* remains low on the priority list of policy makers. Funds are therefore, slow to come by. In the few centers where it has grown, it has remained an offshoot of pharmacology. When it is time to opt between a trained clinician and a clinical pharmacologist for an emerging institute, majority of the times the former wins while the latter struggles for survival. This may sound like a disgruntled voice, well, but a realistic disgruntled voice it is.

Keeping in mind the value a clinical pharmacologist provides in various health related aspects, it is important that these problems are taken care of and a roadmap for increasing the number of centers providing state of the art training in clinical pharmacology is created.

## Concluding Remarks

1. Clinical Pharmacology, though hitherto not a very coveted super speciality subject, is increasingly being opted for by top rankers in entrance exams.
2. Though the number of clinical pharmacologists has declined worldwide, there has been a revival in understanding the importance of training in the subject.
3. Clinical pharmacologists have several important roles to play such as identifying new drug targets, development of new drugs, correct application of available knowledge about drugs, regulatory issues and policymaking regarding therapeutics.
4. In the current era, where the pharmaceutical industry is trying all means to increase their profit (‘me too’ drugs, data dredging, unhealthy promotional practices, concealing unfavourable results, exaggerating potential uses, not declaring conflicts of interests, conducting unethical trials, ignoring the diseases which ail the poor and the majority), a clinical pharmacologist has an important role to play as an informed critic of such practices.
5. Clinical pharmacology provides a platform for collaborative efforts between academia and the pharmaceutical industry. Academia-Industry collaboration would lead to a healthy growth of both academia and industry for the purpose of innovation in health related products, technologies and rational utilization of these facilities in a just manner by all.
6. Certain apprehensions remain regarding the future of clinical pharmacology. These are mainly because of paucity of institutes giving training in clinical pharmacology, shortage of trained faculty, and academia holding against the monetary incentives that the industry offers to clinical pharmacologists. All the same, there are signs of revival of the speciality and it is already beginning to make its presence felt.

### Take Home Message

Clinical pharmacology, though a relatively young specialty, is a branch which is of utmost importance in this era of modern medicine. A clinical pharmacologist with his or her knowledge about various aspects related to new drug development, regulatory requirements pertinent to the same, ethical conduct of clinical trials, interpretation of trial results, conflicts of interest, promotional activities of pharmaceutical companies, neglect of research in diseases afflicting the large majority of population, is a person best suited for providing his or her intellectual input into many drug and therapeutics related aspects.

## Questions That This Paper Raises

1. What is the future of clinical pharmacologists especially in developing countries where the branch is restricted to only a few academic institutes?
2. Will raising the number of clinical pharmacologists improve the quality and quantity of basic and clinical research?
3. Will a good, conscientious rapport between academia and industry contribute towards narrowing the 10-90 gap?
4. What should be the benchmarks for clinical pharmacology units in India and abroad?
5. How can policy makers be made to understand the value of a clinical pharmacologist?

## About the Authors

***Dr Samir Malhotra** is currently working as Assistant Professor in the Department of Pharmacology at Postgraduate Institute of Medical Education and Research (PGIMER), Chandigarh. After doing his M.D in Pharmacology, he passed his D.M, Clinical Pharmacology, from PGIMER, Chandigarh. He has undertaken several basic and clinical research projects, guided M.D, D.M, M.Sc. and Ph.D. theses and organized workshops on Good Clinical Practice. He has published nearly hundred articles in national and international journals. In the field of research, he has been bestowed with several awards. He has served as Editor or sub-editor for medical journals. He has authored a book titled*, ‘All that you wanted to know about Clinical Research.’ *He is Fellow, American College of Clinical Pharmacology, and Member of several other scientific societies*.

***Dr Nusrat Shafiq** has done her M.D. (Pharmacology), D.M. (Clinical Pharmacology) from the Postgraduate Institute of Medical Education and Research, Chandigarh. She is currently working as Senior Research Associate in the same department. She has over thirty national and international publications and is the co-author of the book titled*, ‘All that you wanted to know about Clinical Research.”

During her training in M.D and D.M, she has undertaken several investigatorand sponsor-initiated trials. She is simultaneously pursuing basic science research and is currently working on heat shock proteins. During her training period, she received several best paper presented and published awards. She is Member of American College of Clinical Pharmacology, Indian Pharmacology Society, Association of Physiologists and Pharmacologists of India etc.
